# Modification of Major Contributors Responsible for Latrine Malodor on Exposure to Hypochlorous Acid: The Potential for Simultaneously Impacting Odor and Infection Hazards to Encourage Latrine Use

**DOI:** 10.4269/ajtmh.20-0553

**Published:** 2020-10-15

**Authors:** Tim E. Dennler-Church, Jeremy C. Butz, Joseph E. McKinley, Erika K. Keim, Mary C. Hall, John S. Meschke, JoAnne M. Mulligan, Jeffrey F. Williams, Lori I. Robins

**Affiliations:** 1 Department of Physical Sciences, University of Washington Bothell, Bothell, Washington;; 2 Department of Environmental and Occupational Health Sciences, University of Washington, Seattle, Washington;; 3 Briotech Inc., Woodinville, Washington

## Abstract

Open defecation remains a common practice in developing countries and leads to high incidence and prevalence of acute gastroenteritis, which is most often caused by human noroviruses (human NoV). Encouraging the use of toilets and pit latrines is one method of improving sanitation; however, it is often hindered by not only cultural traditions but also from a reluctance to use latrines and toilets due to their odor and impression of uncleanliness. In an effort to establish new means to encourage toilet and latrine use, laboratory experiments tested the ability of hypochlorous acid (HOCl) to modify the malodorous compounds identified in the air in latrines in developing countries (indole, p-cresol, dimethyl disulfide (DMDS), dimethyl trisulfide (DMTS), and butyric acid) and inactivate MS2 bacteriophage, a surrogate for human NoV. After 5 minutes, > 94% of indole, p-cresol, DMDS, and DMTS was modified as determined by high-pressure liquid chromatography in the presence of 100 ppm HOCl. A log_10_ reduction value (LRV) greater than 6 was seen for MS2 bacteriophage after 5 minutes of exposure to 100 ppm HOCl in solution. Sensory studies indicated that there was a significant difference (*P* ≤ 0.05) between the untreated and HOCl-treated samples for all five malodorous compounds tested. The findings suggest that introduction of HOCl into the headspace air could encourage latrine and toilet use. Optimization of HOCl dosing in air to accomplish both odor control and reduction of infectious hazards is worthy of further study.

## INTRODUCTION

Open defecation remains a common practice in many rural areas of developing countries. The practice contributes to high levels of environmental fecal pathogen contamination and is associated with gastrointestinal disease morbidity that especially afflicts young children ages 1–5 years.^1–3^ Encouraging the construction and use of latrines has become a priority for public health authorities in many regions in an effort to reduce open defecation and improve sanitation in developing countries. Significant philanthropic and government support for these efforts has materialized in recent years.^4–6^ However, despite marked increases in the numbers of installed latrines, rates of adoption for routine use remain low.^7–9^ Widespread aversion to latrine use arises not only from long-standing cultural traditions but also from unwillingness to make use of foul-smelling structures that give rise to the perception of uncleanliness.^10–13^ Odor control clearly needs to be included as part of improving sanitation.

Promoting greater use of latrines in developing countries has the potential to limit transmission of fecal pathogens readily spread via environmental contamination of water and food sources. The perception of uncleanliness of latrines and the suspicions aroused in users about their safety are not entirely misplaced, given the well-established risks of exposure to highly contagious gastrointestinal disease agents, such as human noroviruses (Human NoV), on surfaces exposed to feces and vomitus from infected persons in public toilets.^14–16^ Encouraging higher levels of adoption therefore requires not only remediation of the repulsion generated by malodors but also the introduction of measures offering users assurance of a safe experience by decontamination of the space occupied by latrine users.

Here, we report on laboratory studies aimed at developing an approach to latrine use encouragement that simultaneously addresses the accumulation of mephitic odors, and the need for inactivation of viral pathogens within the superstructure of the facility. The effector chosen is hypochlorous acid (HOCl), a powerful oxidizing and halogenating agent, likely to interact with and disrupt the structure of a wide range of malodorous compounds, and also rapidly inactivate infectious particles.^17–20^ We characterized molecular modifications of the major chemical contributors to latrine malodor identified as p-cresol, dimethyl trisulfide (DMTS), dimethyl disulfide (DMDS), indole, and butyric acid on exposure to HOCl.^11,21^ In parallel, we also demonstrate the feasibility of relying on the antimicrobial efficacy of aerosolized HOCl to bring about meaningful reductions in the infectivity of MS2 bacteriophage particles, as a surrogate for human NoV and other diarrhea-associated viruses.^18,21–24^

## METHODS AND MATERIALS

Reagents for the iodometric titrations were purchased from Hach (dissolved oxygen three powder pillows, potassium iodide powder pillows, sodium thiosulfate digital titrator cartridge, 0.113 N, and starch indicator solution). Sodium chloride (NaCl), sodium thiosulfate (Na_2_S_2_O_3_, STS), potassium chloride (KCl), p-cresol, sodium phosphate, and potassium phosphate were purchased from Fisher Scientific. Water from MilliQ water purification system (MilliporeSigma, Burlington, MA) was used for all experiments. HOCl (pH 4) was provided by Briotech Inc. (Woodinville, WA) as a commercially available end product; higher starting concentrations were used for spray studies, whereas lower starting concentrations were used for solution studies. High-pressure liquid chromatography (HPLC)–grade water and acetonitrile, butyric acid, DMDS, indole, and DMTS were purchased from Sigma Aldrich. Nutrient broth, bacto agar, and tryptic soy agar for *Escherichia coli* and MS2 culture are from BD Difco.

### High-pressure liquid chromatography.

Quantitation of malodor modification by HOCl at various concentration and incubation times was performed by HPLC. The instrument used is a HP Agilent 1100 HPLC with an Eclipse XDB-C18 5 µm 4.6 × 150 mm column (Santa Clara, CA). Each sample had a 1% NaCl background, to maintain consistency with HOCl solutions used. The column temperature was set to 25°C and the flow rate to 1.000 mL/min for p-cresol, indole, DMDS, and DMTS. The flow rate was set to 0.6 mL/min for butyric acid. For p-cresol and indole samples, an isocratic mobile phase of 70/30 water/acetonitrile was used, whereas DMDS and DMTS were run with an isocratic mobile phase of 50/50 water/acetonitrile. An isocratic mobile phase of 0.1% phosphoric acid was used for butyric acid. The injection volumes used were 30 µL for p-cresol, 100 µL for indole, 10 µL for DMTS, 30 µL for DMDS, and 100 µL for butyric acid, whereas the run times for these compounds were 11 minutes, 20 minutes, 12 minutes, and 2.5 minutes, respectively. For both indole and p-cresol, absorbance was measured at 284 nm, whereas the absorbance of DMTS, DMDS, and butyric acid was measured at 240 nm, 263 nm, and 215 nm, respectively. The area under the peak representing each malodor was used to quantitate the extent of modification, and integration parameters were kept consistent within each experiment.

### Modification of p-cresol, indole, DMTS, DMDS, and butyric acid at various HOCl concentrations.

HPLC was used to quantitate the extent of modification of the malodorous compounds. Butyric acid, DMTS, and p-cresol solutions were prepared at 10 ppm. Indole and DMDS solutions were prepared at 50 ppm and 20 ppm, respectively. These solutions had varying concentrations of HOCl, ranging from 0 to 140 ppm, depending on the compound tested. After 5 minutes, the reaction mixtures were quenched with sufficient sodium thiosulfate (STS) to reach a final concentration of 5 mM. Each sample was performed in triplicate. The reaction mixture was analyzed using HPLC as described earlier.

### Residual chlorine determination.

Iodometric titrations were completed to determine if any residual chlorine was present after the reaction of HOCl with the malodorous compounds. Solutions of each malodor were prepared as described in the HOCl concentration reactions (butyric acid, DMTS, and p-cresol solutions were prepared at 10 ppm; indole was prepared at 50 ppm; and DMDS was prepared at 20 ppm) at a volume of 25 mL. The samples were not quenched with STS. After 5 minutes, the samples were titrated following our previously described method.^25^ In brief, iodometric titrations using sodium thiosulfate (0.113 N) were completed using HACH reagent kits for total (active and free) chlorine (Hach Company, Loveland, CO) following HACH method 8,209.

### Gas chromatography mass spectrometry (GCMS).

The products generated by the reaction between the malodorous compounds and HOCl were analyzed by GCMS. For each malodorous compound, 5 mL of 100 ppm malodor was prepared with a concentrated HOCl solution (> 150 ppm) in a 10-mL falcon tube. These reaction mixtures were quenched with sodium thiosulfate (5 mM final concentration) after 10 minutes. The samples were frozen with liquid nitrogen and lyophilized. The resulting powder was suspended in 1.5 mL of absolute ethanol and centrifuged at 16,000 rpm for 1 minute. The supernatant was then filtered through glass wool and analyzed by GCMS (Agilent 6850). The oven was held at 250°C overnight before running the samples to purge out contaminants. Ethanol blanks were run before sampling and in between samples to monitor changes in background contamination. An injection volume of 2 mL was used for each sample, the injector was set to 250°C, and the detector was set to 270°C. The oven temperature was initially 50°C for the first 3 minutes of the run, and then ramped up at a rate of 20°C/minute to 180°C where it was held for 11.5 minutes before the column was purged at 300°C.

### MS2 bacteriophage disinfection.

The efficacy of HOCl against MS2 infectivity was tested using dried MS2 on stainless steel coupons submerged in HOCl and by spray delivery of HOCl. MS2 bacteriophage (ATCC 15597-B1) was prepared by confluent lysis on *E. coli* F-amp (ATCC 70081) in 15 mL soft agar (0.5% Bacto agar and 0.7% NaCl) poured onto a nutrient agar petri dish and incubated at 37°C overnight. Propagated MS2 was further extracted via organic solvent extraction using Vertrel XF (Dupont, Wilmington, DE) by vigorously mixing scraped top layer of soft agar and equal volumes of Vertrel XF followed by centrifugation at 3,500 × *g* at 4°C for 15 minutes. Centrifuged supernatant was collected and stored in 1 mL aliquots at −80°C. All MS2 stocks and samples were quantified by 10-fold serial dilutions and plating using the double agar layer (DAL) method as previously described.^26^

### MS2 bacteriophage disinfection using stainless steel coupons submerged in HOCl.

Stainless steel coupons (BioSurface Techologies Corp., Bozeman, MT) were seeded with ∼10^6^ of MS2 stock that had been diluted in phosphate-buffered saline (PBS) and filtered using a 0.1 µm polyvinylidene difluoride (PVDF) membrane filter and dried for 1 hour in a biosafety cabinet (BSC) or fume hood. Discs were transferred to 20 mL high-density poly ethylene Teflon-coated Wheaton^®^ scintillation vials (Millville, NJ) and submerged with 2 mL of Briotech HOCl at various concentrations for a period of 5 minutes or at 50 ppm HOCl with varied contact times at 20°C. At desired contact times, all MS2 seeded discs were quenched using PBS with 2 mL of 1% sodium thiosulfate and eluted off discs via 4 minutes of vortexing at max speed. Samples were serially diluted and quantified using DAL method and compared with reference controls to determine the plaque-forming unit (PFU) reduction as a basis of HOCL disinfection. Each experiment was repeated 2–3 times and included at least three reference controls to help estimate and account for experimental losses and variability. Each experimental variable contained 3–-6 biological replicates.

### MS2 bacteriophage disinfection using stainless steel coupons with spray delivery of HOCl.

Stainless steel coupons (BioSurface Techologies Corp.) were seeded with ∼10^6^ of MS2 stock that had been diluted in PBS and filtered using a 0.1-µm PVDF membrane filter and dried for 1 hour in a BSC or fume hood. To determine the effect of the disinfectant via spray delivery on MS2, HOCl (260–360 ppm) in a standard spray bottle was set up at a 45° angle at a height of 10 cm to deliver disinfectant 15 cm from the nozzle. HOCl spray bottles were primed before delivery onto MS2-seeded stainless steel discs with a contact time of 5 minutes. Disinfectant dosage varied based on the number of sprays applied to stainless steel discs. At desired contact times, all MS2-seeded discs were quenched using PBS with 2 mL of 1% sodium thiosulfate and eluted off discs via 4 minutes of vortexing at max speed. Samples were serially diluted and quantified using DAL method and compared with reference controls to determine the PFU reduction as a basis of HOCl disinfection. Each experiment was repeated 2–-3 times and included at least three reference controls to help estimate and account for experimental losses and variability. Each experimental variable contained 3–6 biological replicates.

### Sensory testing for difference.

Sensory tests were performed to determine if there was a sensory difference between malodorous samples treated with HOCl and those without HOCl. Purified deionized water was used for palate cleansing to clear the sense of smell during the sensory evaluations. Deionized water was filtered over a Milli-Q reagent water system containing carbon, deionizing, and trace organic filters (Millipore, Bedford, MA). The 20 mL disposable scintillation clear vials were purchased from WSU Central Stores, WA. NaCl was purchased from J.T. Baker Co (Phillipsburg, NJ).

One day before the panel, all aroma stock solutions were prepared. For these preparations, 200 µL of stock aroma solutions was mixed with 1800 µL of 1% NaCl (resulting in a 50-ppm solution)—this was defined as the control solution. The intervention solutions were prepared on the day of the panel, no more than 1 hour before presentation to the consumer. For these solutions, a 200-µL stock aroma solution was mixed with 1800 µL intervention solution (150 ppm HOCl), resulting in a 50-ppm solution. All vials were labeled with three-digit codes and maintained in the dark once prepared.

The sensory panel for difference testing of the five chemical compounds was conducted on day 1. The panel was composed of 80 untrained participants (consumers). The gender demographics of the consumers are described in Table 1. The average age was 36 years. Consumers were recruited from the Washington State University community, as well as the broader Moscow-Pullman, Washington community. A minimum amount of information on the nature of the study was provided to reduce potential bias. The project was approved by the Washington State University Institutional Review Board. Each consumer was presented with a nonmonetary incentive for their participation.

**Table 1:** Sensory triangle difference test

	Butyric acid	p-cresol	DMDS	DMTS	Indole
Responses	Treated vs. untreated				
Correct	65*	62*	73*	64*	63*
Incorrect	15	18	7	16	17
Total	80	80	80	80	80

DMDS = dimethyl disulfide.; DMTS = dimethyl trisulfide.

* Significance at *P* ≤ 0.05.

Number of correct responses for triangle difference test of butyric acid, p-cresol, DMDS, DMTS, and Indole (*n* = 80). Untreated samples were 50 ppm solutions in 1% NaCl; treated samples were 50 ppm solutions in 135 ppm hypochlorous acid. The gender composition of the panel was 35% male and 65% female.

In the triangle difference test, two samples were the same, and one is different. Consumers were required to select the most different sample from this set of three samples and provide comments about the difference observed. All consumers received all five aroma compounds, resulting in five flights of samples being evaluated by each consumer. Samples were served as 2 mL solutions in 20 mL glass vials. Samples were presented in a random order under white light. Consumers were asked to pause for 30 seconds between sample evaluations to refresh their olfactory senses. Statistical analysis was performed in Compusense according to the method of Roessler et al.^27^ The level of significance for treatment differences was established at *P* < 0.05.

## RESULTS

**Figure 1.**
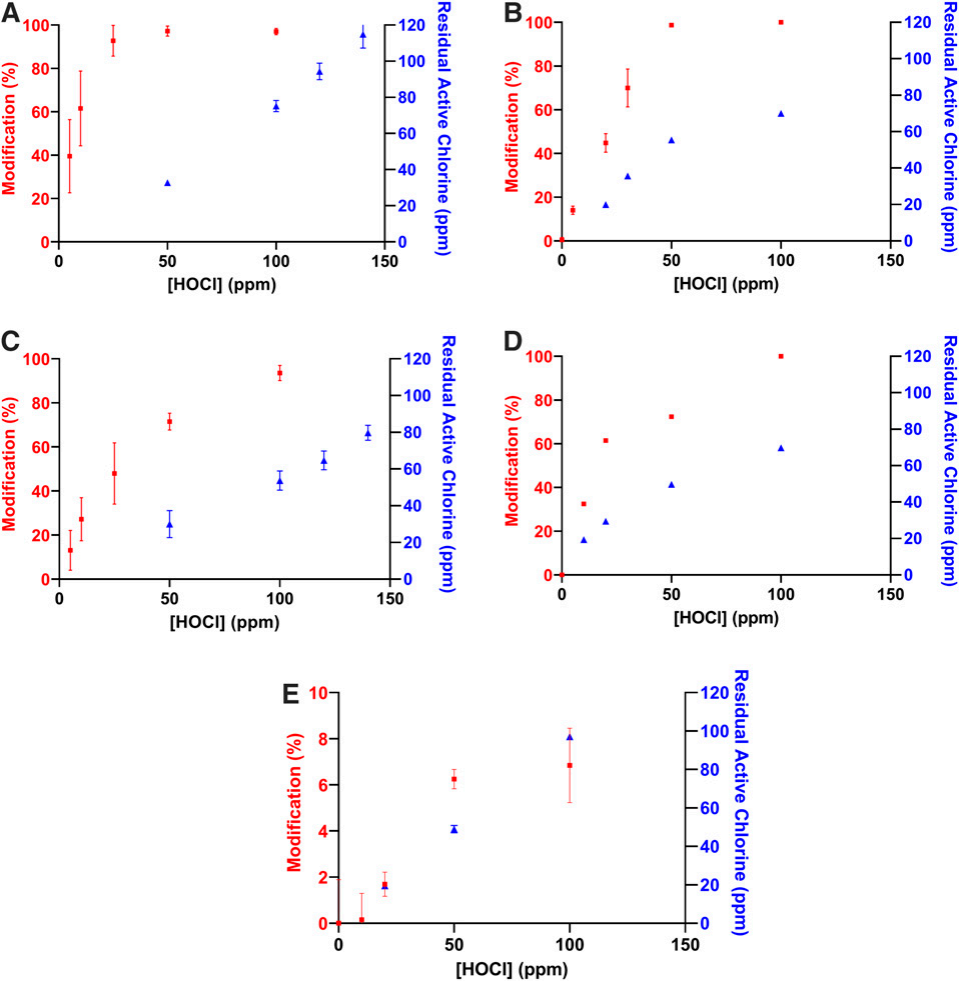
Elimination and residual active chlorine for p-cresol, dimethyl disulfide (DMDS), dimethyl trisulfide (DMTS), indole, and butyric acid. (**A**) 10 ppm p-cresol with various concentrations of hypochlorous acid (HOCl). (**B**) 50 ppm indole with various concentrations of HOCl. (**C**) 10 ppm DMTS at various concentrations of HOCl. (**D**) 20 ppm DMDS at various concentrations of HOCl. (**E**) 10 ppm butyric acid at various concentrations of HOCl. All samples were incubated for 5 minutes; the percent elimination was determined by high-pressure liquid chromatography after quenching with sodium thiosulfate, and the residual active chlorine was determined by iodometric titrations. Red = % modification; blue = residual chlorine (ppm). This figure appears in color at www.ajtmh.org.

**Figure 2.**
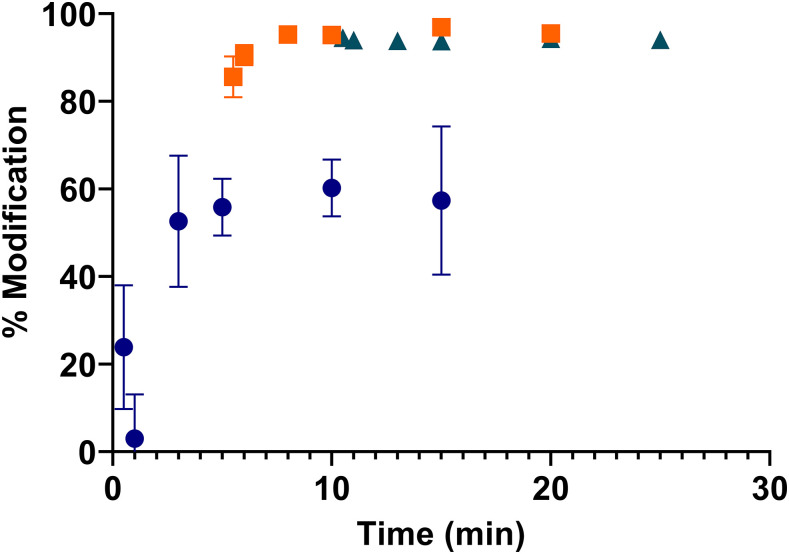
Sequential addition of hypochlorous acid (HOCl) to a solution of 10 ppm p-cresol. Blue circles = first addition of 10 ppm HOCl; orange squares = second addition of 10 ppm HOCl; green triangles = third addition of 10 ppm HOCl. This figure appears in color at www.ajtmh.org.

High-pressure liquid chromatography was used to quantitate the modification of the key odorants in latrine headspace. 10 ppm p-cresol solutions were reduced by > 97% at concentrations of HOCl greater than 50 ppm (Figure 1), with the reaction reaching completion within 5 minutes. Sequential additions of low concentration HOCl (10 ppm) were added to the p-cresol solution (10 ppm) in an attempt to increase the percent modification. After a second addition of HOCl, > 95% p-cresol was modified; no additional modification was seen after a third addition of HOCl (Figure 2). Indole was more readily modified by HOCl than p-cresol. Concentrations of 50 ppm HOCl modified > 98% of indole (50 ppm); at 100 ppm HOCl, remaining indole was undetectable by HPLC within 60 seconds. Dimethyl disulfide (20 ppm) and DMTS (10 ppm) both reacted readily with HOCl, although DMTS appeared less reactive which may be due to residual ethanol used for solubility. Dimethyl trisulfide (> 93%) was modified in the presence of 100 ppm HOCl, whereas DMDS was not detectable by HPLC after the addition of HOCl at concentrations greater than 50 ppm (Figure 1). Butyric acid was the least reactive compound tested showing only ∼6% modification in the presence of 100 ppm HOCl after 5 minutes; after longer periods of time, no further modification was seen (see Supplemental Information).

Indole, p-cresol, and DMDS followed similar trends with respect to residual chlorine concentrations. For these compounds, the residual chlorine concentration was found to increase proportionally to the amount of HOCl added after the malodor was modified to the extent that it was no longer detectable. Dimethyl trisulfide, in contrast, did not follow this same trend, showing a presence of free chlorine well before full modification of the malodor was achieved. Free chlorine concentrations showed little, if any, change in the presence of butyric acid.

Sensory triangulation tests were carried out to determine if the analytical data were supported by the human senses. A significant difference (*P* ≤ 0.05) between the untreated and treated samples with all tested samples, including butyric acid (Table 1), was found from consumer tests.

**Figure 3.**
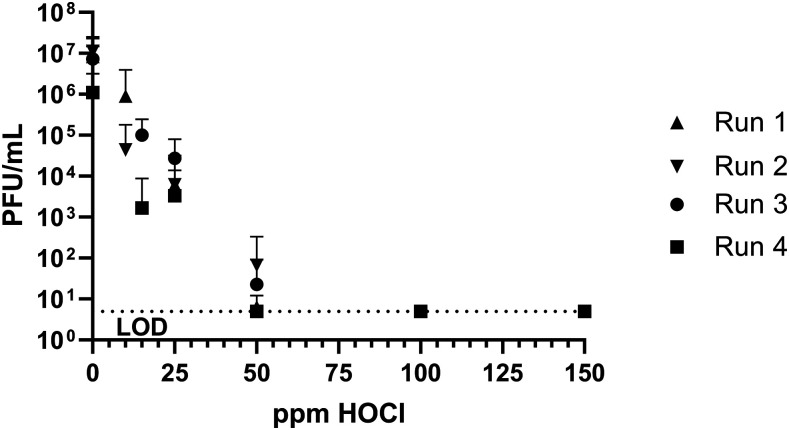
MS2 bacteriophage PFU/mL after 5 minutes of coupon submersion with various concentrations of hypochlorous acid.

**Figure 4.**
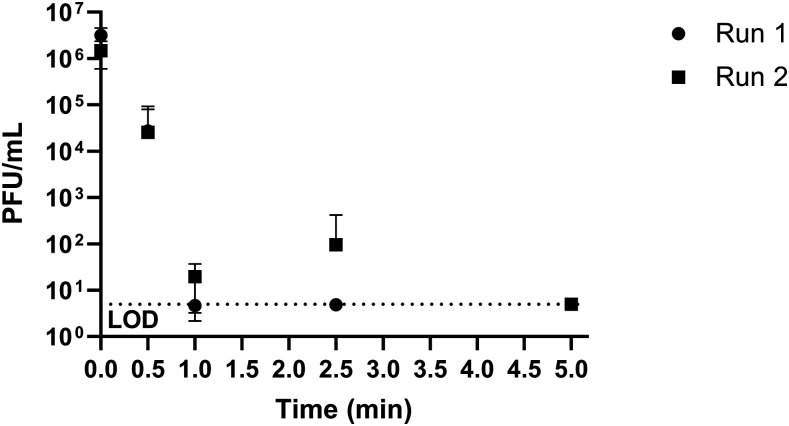
MS2 bacteriophage PFU/mL after various times of coupon submersion with 50 ppm hypochlorous acid.

Inactivation of dried MS2 bacteriophage on stainless steel coupons by HOCl was tested as a surrogate for human NoV to determine effective concentrations and contact times, and demonstrate disinfection via aerosol delivery. A log_10_ reduction value (LRV) > 5.6 was seen with HOCl concentrations greater than 50 ppm at 5 minutes of contact time (Table 2, Figure 3). Even at low concentrations of HOCl (15 and 25 ppm), a significant decrease in culturable MS2 plaques was seen (Table 2). Reductions of culturable PFUs were seen as quickly as 30 seconds of contact time during time course experiments, with the most effective contact time being 5 minutes (Table 3, Figure 4). Higher concentrations of aerosolized HOCl (240 ppm) also inactivated MS2 bacteriophage on stainless steel coupons where increasing the number of sprays applied led to increased MS2 disinfection (Table 4).

**Table 2:** Change in MS2 plaque formation after coupon submersion with various concentrations of hypochlorous acid and 5 minutes contact time

Treatment (*n*)	Mean log_10_ ± 95% CI	σ_*x*_	Log_10_ reduction value ± 95% CI
0 ppm (12)	6.6 ± 0.28	0.49	–
10 ppm (6)	4.8 ± 1.0	1.2	1.9 ± 1.0
15 ppm (6)	3.3 ± 1.7	2.1	3.3 ± 1.7
25 ppm (12)	2.9 ± 0.89	1.6	3.7 ± 0.93
50 ppm (12)	0.95 ± 0.29	0.52	5.7 ± 0.40
100 ppm (9)	< 0.70 ± 0.0	0.0	> 6.0 ± 0.28
150 ppm (6)	< 0.70 ± 0.0	0.0	> 6.0 ± 0.28

**Table 3:** Change in MS2 plaque formation based on contact time of coupon submersion in 50 ppm hypochlorous acid

Time point (*n* = 6 for all) (Minutes)	Mean log_10_ ± 95% CI	σ_*x*_	Log_10_ reduction value ± 95% CI
0	6.3 ± 0.16	0.20	–
0.5	3.9 ± 1.0	1.3	2.4 ± 1.0
1	1.0 ± 0.28	0.35	5.7 ± 0.32
2.5	1.1 ± 0.57	0.71	5.2 ± 0.59
5	< 0.70 ± 0.0	0.0	> 5.6 ± 0.16

**Table 4:** Change in MS2 plaque formation due to spray delivery of 240 ppm hypochlorous acid at a distance of 15 cm

Number of sprays (*n* = 3 for all)	Mean log_10_ ± 95% CI	σ_*x*_	Log_10_ reduction value± 95% CI
0	6.6 ± 0.33	0.3	–
5	5.4 ± 0.67	0.6	1.2 ± 0.74
15	1.8 ± 1.8	2.0	4.8 ± 2.3
25	< 0.70 ± 0.0	0.0	> 5.9 ± 0.33
50	< 0.70 ± 0.0	0.0	> 5.9 ± 0.33

## DISCUSSION

Hypochlorous acid can alter most of the compounds responsible for the latrine odor with changes revealed by both chemical and sensory analyses. It is well established that HOCl can act through chlorination and oxidation reactions to modify various classes of biomolecules, compounds, and functional groups including thiols, amines, aromatic amino acids, and peptide bonds.^17,28,29^ For example, oxidation of thioethers (e.g., methionine) leads to the formation of sulfoxides, whereas the chlorination of tyrosine leads to the formation of both 3-Cl-tyrosine and 3,5-dichlorotyrosine; radical formation can lead to tyrosine dimerization.^29,30^ Our results align well with these studies and indicate that HOCl is able to modify, even to a small extent (i.e., butyric acid), the major sources of malodor found in latrines in developing countries (Figure 1). As expected, analysis of the products by GCMS identified 2-chloro-4-methyl-phenol and 2,6-dichloro-4-methyl-phenol from p-cresol.^31–34^ Oxidized products of indole include indole-2-one (oxindole) and 1H-indole-2,3-dione (isatin).^35,36^ Products of DMDS and DMTS in the presence of HOCl include sulfonyl chlorides and thiosulfonate esters, as expected (see Supplemental Information).^37^ With the small amount of modification to butyric acid treated with HOCl, it was not surprising that products were not identifiable by GCMS.

Reactions with most of the malodor compounds were complete within 5 minutes, indicative of the practical usefulness of HOCl as an intervention. For example, a 1.7-m^3^ latrine used to assess perfume performance on latrine malodors would be expected to contain, under the dirtiest conditions, 2.6 μg of indole and 15.3 μg of p-cresol based on previous GCMS headspace analysis.^21,38^ These masses would require less than 2 mL of aerosolized HOCl solution (100 ppm) for > 95% modification based on our chemical analysis studies. Similarly, the concentrations of DMDS and DMTS tested were > 2,000 times higher than the odor detection threshold from the headspace of ethanol/water solutions at concentrations of 9.1 and 0.4 μg/L, respectively.^39^ This suggests that the concentration of HOCl used in these studies would be more than sufficient for the intended use to combat latrine odor.

Hypochlorous acid concentrations of ∼100 ppm brought about significant changes to p-cresol, indole, DMTS, and DMDS using solution concentrations of the malodor compounds, ranging from 10 to 50 ppm in 5 minutes (Figure 1). At this same concentration and time, > 6.0 LRV of MS2 bacteriophage was seen with coupons submerged in HOCl (Table 2); a similar log reduction value of MS2 bacteriophage was seen with coupons submerged in 50 ppm HOCl for 5 minutes (Table 3). Spray delivery of HOCl (initial: 240 ppm, delivered: ∼160 ppm) to coupons gave comparable results after > 25 sprays as seen in Table 4. Taken together, these studies provide measurable and reproducible results that allow for quantifying the effects of HOCl against both MS2 and latrine malodors in solution and/or by spray delivery. Furthermore, this study sets the foundation for understanding the effectiveness of aerosolized HOCl deployed into the latrine superstructure airspace against mixtures of malodors and virus particles. It provides a basis for a realistic opportunity to encourage latrine use by amelioration of malodor in the latrine superstructure, and also significantly decreases the potential for contamination and transmission of gastrointestinal viral pathogens including human NoV.

The sensory analysis of p-cresol, indole, DMDS, and DMTS further supports the analytical chemistry for p-cresol, indole, DMDS, and DMTS, indicating that treatment with HOCl led to a change detectable by the human senses for these compounds. Unexpectedly, the sensory data indicated this was also the case for butyric acid, whereas the analytical data showed very little change. This could be due to potential masking of the odor with the HOCl, giving a slight chlorine smell according to panel comments (see Supplemental Information). Some panelists described this same scent for changes with treated and untreated samples of p-cresol, indole, DMTS, and DMTS; others did not report a chlorine scent, but detected a change in the scent (see Supplemental Information). Changes to the analytical and sensory profiles for the more reactive compounds (p-cresol, indole, DMDS, and DMTS) are most likely due to oxidation and chlorination reactions changing the concentration of the malodorants in the headspace; masking is most likely for butyric acid samples. It is well documented that modification of one compound affects the sensory perception of the overall mixture, and it is therefore likely that HOCl will improve latrine odor; however, further testing with mixtures of these specific malodorants will be needed to confirm that supposition.^40,41^

It is clear from this exploratory study that aerosolized HOCl has the potential to encourage wider adoption of latrine use by improving or eliminating off-putting smells in latrine superstructures and reducing hazards associated with enteric viruses in the air and on the surfaces of latrines. Odor control in latrines has recently been brought to the world stage with a variety of collaborations including one between the Bill & Melinda Gates Foundation project Reinvent the Toilet Challenge and Firmenich SA that has successfully identified the compounds responsible for the odors in latrines and developed perfume to improve the overall latrine environment.^11,21,38^ Hypochlorous acid offers an opportunity for an alternative intervention that could significantly modify most of the identified malodor compounds, and therefore the sensory perception, and simultaneously bring about improved sanitation within the latrine environment. HOCl as an alternative offers stability at ambient and elevated temperatures, potency, low cost, and the prospect of modifying noxious chemical compounds and inactivating infectious microbes.^25,42^ Aerosolized HOCl has been used in a variety of applications to control infections (e.g., livestock and hospitals), and passive deployment technologies (e.g., motion detecting air-freshener dispensers) are available that are low cost, making it possible to dispense HOCl in a controlled way for use in developing countries.^24,43,44^

Further work on this project as it moves into the next phase will involve studying the dose and time response of aerosolized HOCl in the modification of the malodor compounds and against human NoV to confirm that the deployed concentration is effective for simultaneous odor control and sanitation as well as sensory tests for the mixtures of the malodor compounds. Encouraging the use of latrines and reducing open defecation in developing countries are critical to reduce unacceptably high rates of gastrointestinal disease. This is a challenge that has been difficult to overcome; however, simultaneous odor control and pathogen reduction have the potential to stimulate wider adoption of latrine use.

## Supplemental tables and figures

Supplemental materials
