# Supplementary material for: Modification of Major Contributors Responsible for Latrine Malodor on Exposure to Hypochlorous Acid: The Potential for Simultaneously Impacting Odor and Infection Hazards to Encourage Latrine Use
Source: Am J Trop Med Hyg. 2020 Oct 15;103(6):2584–90. doi: 10.4269/ajtmh.20-0553 (PMC7695106; doi:10.4269/ajtmh.20-0553)
Supplement: Supplementary file 1 [file tpmd200553.SD1.docx]

# **Supplemental Figure S1.** Time course data for butyric acid (50 ppm) with HOCl (250 ppm) monitored by HPLC.

**Supplemental Figure S2.** GCMS traces of malodorous compounds. Top to bottom: p-cresol, indole, DMDS, DMTS, and butyric acid. Blue = before addition of HOCl; Orange = after addition of HOCl (orange). The products of the reactions are labeled.

**Supplemental Table S1.** Comments of panelists on why they chose the sample (butyric acid treated or untreated) to be different in the triangle test. These comments are only from those consumers who correctly identified the different sample.

| Panelists # | Comments | Panelist # | Comments |
| --- | --- | --- | --- |
| wsuguest48 | 180 and 896 didn't really smell like anything | wsuguest14 | The other two smelled like a swimming pool, and 180 had no smell. |
| wsuguest70 | They all smell bad, but 449 smells worse, and has more of a chlorine scent. | wsuguest38 | This had no smell |
| wsuguest65 | I couldn't smell the other two, and 143 smelled like old pool water | wsuguest75 | It didn't have a smell that I could percieve while the other 2 smelled of pool water |
| wsuguest60 | much stronger and stimulating than the other two | wsuguest44 | 449 and 143 smell kind of rotten |
| wsuguest34 | the first two had almost no aroma, but this sample had a chlorine-like aroma. | wsuguest63 | It basically had no smell compared to 143 and 449 |
| wsuguest42 | the other two didn't smell like much | wsuguest56 | it is the smooth one |
| wsuguest17 | much stronger odor | wsuguest31 | no aroma |
| wsuguest76 | pungent smell as opposed to no smell | wsuguest61 | Scent is barely detectable. |
| wsuguest53 | samples 896 and 180 had little to no distinguishable aroma | wsuguest15 | They all smelled of cheese but the first one was incredibly weak. |
| wsuguest35 | Seemed more chemical cleaner smell but very subtle | wsuguest13 | more neutral smell than the other two |
| wsuguest4 | this sample did not smell anything like the first two samples. | wsuguest27 | Honestly this one was REALLY hard! They all smelled like mold - like my electric toothbrush when I don't clean it for a while, haha! Or maybe cheese when it is starting to ripen. But 143 and 449 had the strongest smell of cleaning fluid or chlorine. 896 was hard to smell in general - I really had to get my nose in there, and I only really got the faint aroma of mold. I also got a tiny bit of floral on there. |
| wsuguest29 | it has the smell swimming pool water with chlorine whereas rest both has not sensible aroma | wsuguest80 | It had no smell |
| wsuguest66 | the other two samples barely had any odor to them, but sample 449 had a very strong bleach/chemical smell to it. | wsuguest21 | 896 has no odor that I can detect, the other two do |
| wsuguest10 | The other two samples almost didnt have any samples. while this sample had a very potent smell. | wsuguest3 | the least potent |
| wsuguest52 | 180 and 896 have fruity smell | wsuguest49 | This sample had no smell in it, while the other two samples had some impurities. |
| wsuguest16 | The other two had a slightly sweet note that 449 didn't have. 449 smelled more like a cleaning product or chlorine. | wsuguest26 | The other two smelled like bleach, while 180 didn't smell like anything. |
| wsuguest28 | The other two were very mild aroma. This one was stronger. | wsuguest73 | The other two samples smelled strongly of bleach, this one did not |
| wsuguest59 | It had strong aroma. | wsuguest20 | No smell. |
| wsuguest5 | the other two are very mild almost no smell and this one smells like bleach | wsuguest2 | 449 and 143 smell like roses |
| wsuguest47 | Samples 896 and 180 smell like water. Sample 143 smells like water with a little bit of chlorine added. Kind of smells like a swimming pool locker room. | wsuguest8 | this one did not have a smell |
| wsuguest36 | strongest aroma | wsuguest39 | the aroma is quite moderate not quite concentrated, need to sniff hard to identify, quite like cat-pee............ |
| wsuguest24 | 449 was awful, the other two I didn't really notice anything. | wsuguest25 | It had no aroma. The other two vials had a slight aroma to them, but not 896. My nose picked up nothing |
| wsuguest41 | Sample smelled different, had a strong odor | wsuguest23 | this one smelled like a toilet. the other 2 did not |
| wsuguest22 | Seemed more acrid, sort of ammonia or chlorine. Others seemed very faint and neutral | wsuguest50 | Had no smell. |
| wsuguest12 | This is the only sample that had an aroma. | wsuguest55 | No smell |
| wsuguest58 | while the other two samples had very little if any odor at all, 449 smelled terrible. | wsuguest57 | little or no oder |
| wsuguest71 | Is the only one you can perceive an aroma on, it is slightly bleachy | wsuguest45 | 896 smelled like nothing where as 449 smelled like buteric acid and 143 smelled like chlorine and buteric acid |
| wsuguest18 | sharp bad smell | wsuguest9 | this sample has no smell |
| wsuguest46 | It was the only one with a scent; the other two did not have an aroma. | wsuguest62 | aroma not so strong |
| wsuguest54 | the other two had no scent at all | wsuguest7 | 896 smelled like nothing at all!!! it was like sniffing water. The other two smelled like chlorine and crayons. |
| wsuguest64 | other two were bland aroma, but 449 has some FUNK | wsuguest33 | The sample 896 has a less pungent odor. |
| wsuguest30 | stronger. smelled bleachy | wsuguest79 | different |

**Supplemental Table S2.** Comments of panelists on why they chose the sample (p-cresol treated or untreated) to be different in the triangle test. These comments are only from those consumers who correctly identified the different sample.

| Panelist # | Comments | Panelist # | Comments |
| --- | --- | --- | --- |
| wsuguest35 | Can't put it into words | wsuguest11 | This sample has a light citrus kind of smell while the others are more earthy and have a stronger base aroma. |
| wsuguest71 | Very different aroma, it smells like plastic, artificial | wsuguest54 | smelled like salt water and the others had a more animal/barn smell |
| wsuguest46 | The first two almost had a floral aroma; sample 285 had a chemical aspect to it. | wsuguest72 | this one was more burnt plastic than the other two |
| wsuguest64 | the other two smell like barn | wsuguest73 | This one smelled like manure/grass hay as opposed to the chlorine/bleach smell of the other two |
| wsuguest30 | stronger odor | wsuguest32 | did not have a sweet smell as the others |
| wsuguest16 | Had some trouble differentiating between these samples but 285 seems a little different from the other 2. | wsuguest49 | It didn't have Chlorine smell in it, while the other two samples had chlorine smell in it. |
| wsuguest4 | this sample did not smell even close to the previous two samples. | wsuguest43 | * |
| wsuguest34 | 285 had a pungent alcohol-like smell to it. | wsuguest26 | This one smells like glue while the other two smell like wet freshly cut grass. This 976 one was a less pleasant smell than the other two. |
| wsuguest10 | It has a much more potent and strong smell than the other two | wsuguest50 | It smelt slightly different then the other two. |
| wsuguest29 | rest two has aromas associated with biomass I guess whereas 297 isn't | wsuguest75 | it smelled like a petting zoo where as the other 2 smelled like pool water |
| wsuguest70 | 285 smells slightly chlorinated, though it still has the same base notes as the other two, which is kind of a burnt rubber scent. | wsuguest39 | recall horse sweep, other two more like hospital theme |
| wsuguest65 | The other two smelled like barnyard animals, while 297 smelled like old pool water. | wsuguest15 | It smelled musky while the others smelled like a cleaner. |
| wsuguest23 | smelled more like chlorine than the other 2 | wsuguest69 | the smell is different |
| wsuguest17 | it had a sharper smell | wsuguest27 | This one had a very floral scent to it. The other two were very reminiscent of chlorine and sulfur. I got a little bit of floral from 297, but the chlorine/sulfur smell really outweighed it. |
| wsuguest22 | This one seemed a bit more swimming pool. Others were both sort of hay/musty. | wsuguest3 | i felt it was the most "dull" or less potent |
| wsuguest76 | chlorine smell as opposed to a rubber smell | wsuguest1 | a cooked cleaner |
| wsuguest78 | it is stronger | wsuguest8 | this one smelled like bandaid, others like chlorine |
| wsuguest5 | there was a different stronger smell with this one | wsuguest56 | it is most strong and pungent |
| wsuguest77 | i smell it differently from others. the others seem different in intensity for me. | wsuguest21 | 899 does not appear to have much of an odor, the other two do |
| wsuguest36 | slightly different aroma | wsuguest80 | It smelled more like horse than chlorine |
| wsuguest47 | Sample 297 has a sharper aroma. Smells more like bleach than the other two, I believe. | wsuguest63 | 899 had a smell but it was different than 297 and 285 (which were very similar) |
| wsuguest42 | strong chlorine smell | wsuguest44 | 976 smells similar, but not quite as potent. It's hard to tell the difference though because the smells seem to linger |
| wsuguest60 | It's more stimulating than the other two. | wsuguest74 | it smells like cough syrup and the other two smell like methane |
| wsuguest28 | It had a stronger, sharper aroma. | wsuguest20 | Band-aid smell |
| wsuguest40 | 285 is strong and having different aroma than other two samples | wsuguest68 | It smelled nice, and not like an overly clorinated pool. |
| wsuguest12 | This sample burned my nose more then the other two samples. | wsuguest7 | They all smelled mostly the same, like that iodine disinfectant, but 899 was much more subtle |
| wsuguest52 | 285 smells different | wsuguest31 | no aroma |
| wsuguest24 | It smells more like chlorine the other two remind me of a medical office. | wsuguest19 | While 297 and 285 smelled harsh and maybe "astringent", 899 was maybe a muskier aroma. I'm not sure how to define it. |
| wsuguest53 | 297 smelled like bleach, 899 and 976 smelled like bandaids | wsuguest37 | This sample was not as strong as the other two and much more bearable. The first 2 were so strong that I didn't want to take that third "sniff". |
| wsuguest79 | It smells different | wsuguest9 | this is more like ink type smell |
| wsuguest55 | It has animal dung/urine smell | wsuguest51 | It smells like leather, rather than just chemical...more natural |

**Supplemental Table S3.** Comments of panelists on why they chose the sample (dimethyl disulfide treated or untreated) to be different in the triangle test. These comments are only from those consumers who correctly identified the different sample.

| Panelist # | Comments | Panelist # | Comments |
| --- | --- | --- | --- |
| wsuguest53 | smelled like bleach, the other two did not. They smelled rotten | wsuguest72 | this one was light pool smell and the other two were broccoli |
| wsuguest77 | it smells different from others. | wsuguest42 | it didn't smell like garlic |
| wsuguest22 | 470 was somewhat chlorinated. The other two were very stinky and sour; not pleasant at all. | wsuguest75 | 479 and 439 smell like pool water to me where as 452 smelled of garlic powder/salt |
| wsuguest46 | The first two were pungent; 470 barely had an aroma. | wsuguest15 | It smells like barbeque sauce and the others smelled like baking soda. |
| wsuguest71 | It smells very different from the other two, it smells like bleach | wsuguest8 | the other two smelled like chlorine. this one smelled saltier |
| wsuguest11 | There is only a faint aroma associated with this sample which makes me think of a clean scent while the others smell like a cooking seasoning. | wsuguest51 | strong compared to others. the others are faint. sample 452 smells like a pre-emergent pesticide I used to apply. it prevented germination. |
| wsuguest48 | It smelled like bleach and the other smelled like cooking asparagus | wsuguest56 | it is the most strongest |
| wsuguest41 | sample had a different smell | wsuguest20 | Does not smell like bleach |
| wsuguest65 | 439 smelled like old pool water while the other two smelled like stinky onions | wsuguest25 | The other 2 samples barely had any aroma, but 452 was foul. It hit me immediately, like a nasty garbage truck full of putrid material |
| wsuguest34 | 470 had a burning-like smell to it. | wsuguest61 | Strong garlicy aroma. |
| wsuguest16 | 470 smelled more chemical like. | wsuguest27 | 452 smelled like a combination of buttered popcorn and cooked vegetables. I got almost nothing from 439 except cleaning solution/chlorine and a little alcohol; while 470 was a stronger version of 439 - it even burned my nose a little bit! So, 470/439 were very alcoholic/sanitizing/cleansing solution, while 452 had more food smell (butter and vegetables). I got no alcohol from that one. |
| wsuguest40 | aroma is 470 is entirely different than 573 and 452 | wsuguest13 | it smelled different than the other two samples |
| wsuguest4 | this sample did not smell like the previous 2 samples. | wsuguest26 | All of them smell quite sharply but 573 was the strongest, and had a sharp garlic and sweat smell to it. Not very pleasant. |
| wsuguest54 | 470 smelled the least aweful | wsuguest9 | 452 has more like garlic type smell whereas other two samples has more like chlorine water that we get in swimming pool type smell |
| wsuguest36 | less strong | wsuguest33 | highest pungency among the rest. |
| wsuguest23 | the other two smelled like someone's breath that ate garlic, this one smelled like chlorine | wsuguest37 | Stronger scent that was very offputting |
| wsuguest59 | strong smell | wsuguest79 | the other two smelled like bleach |
| wsuguest5 | it just smell different and not nearly as strong and pungent | wsuguest7 | 452 smelled oniony and like a chemistry lab. the other two smelled very lightly of chlorine |
| wsuguest10 | The other two smelled like a food sample while this sampled smelled like water that sat in the sun for a long time or water that isnt good to drink from | wsuguest38 | Other 2 smelled like HCl |
| wsuguest78 | it has a scent of clorine | wsuguest67 | The first two were bleachy and the last one more salty. |
| wsuguest70 | 470 smells like chlorine while the other two smell like...garlic? | wsuguest14 | 573 was the most different, with a kind of rotting smell that made it more distinct. |
| wsuguest35 | More neutral odor than other two | wsuguest44 | 470 and 439 smell kind of like natural gas- 573 was awful |
| wsuguest30 | stronger smell--Bad! kind of putrid | wsuguest62 | much more intense aroma |
| wsuguest47 | This sample has less of a bite than the other two. Sample 439 smells like of like armpit meets chlorine. The other two have kind of a savory aroma. | wsuguest57 | spice note |
| wsuguest6 | The other 2 samples had a much more garlic smell | wsuguest80 | It smelled like onion powder instead of chlorine. |
| wsuguest12 | Sample 470 has a very different smell then the other samples. | wsuguest73 | The other two samples smelled like bleach, this one smelled like garlic or onion |
| wsuguest17 | less sharp smell | wsuguest43 | the other two smelled like chlorines |
| wsuguest76 | chlorine smell as opposed to a sulfur smell | wsuguest55 | Very strong chemical smell (like cooking gas) |
| wsuguest58 | 470 again smelled horrible like some sort of fuel while the other two samples 573 and 452 smelled more like grilled brussel sprouts | wsuguest49 | The first two sample had more chlorine like smell, while this sample had different smell than chlorine. |
| wsuguest29 | it has a typical pungency than others | wsuguest19 | It smelled sweeter and not as harsh as the other two samples. The first two samples smelled the same to me except that 470 seemed stronger. |
| wsuguest64 | most unique | wsuguest39 | first one remind me of some painting oil. |
| wsuguest52 | 470 still smells like chlorine | wsuguest45 | smelled like meat/spices where as the others smelled like chlorine |
| wsuguest68 | It smelled like rotting broccoli, instead of a pool. | wsuguest31 | stronger |
| wsuguest2 | 573 smells like onions | wsuguest32 | only sample with detectable odor |
| wsuguest50 | Strong chemical smell | wsuguest63 | 452 was vomit inducing |
| wsuguest1 | spicey green pepper obvious | wsuguest74 | it smells like black pepper and the other two smell like chlorine and water |
| wsuguest21 | all three have odors but 452 appears very distinctive |  |  |

**Supplemental Table S4.** Comments of panelists on why they chose the sample (dimethyl trisulfide treated or untreated) to be different in the triangle test. These comments are only from those consumers who correctly identified the different sample.

| Panelist # | Comments | Panelist # | Comments |
| --- | --- | --- | --- |
| wsuguest17 | less of an egg odor | wsuguest49 | This sample had stronger smell the other two samples. |
| wsuguest47 | Samples 630 and 691 smell savory and meaty. The smell is familiar but I am having trouble identifying exactly what that smell is. Sample 541 smells like a chemical product. | wsuguest21 | all three have odors but 630 appears to be very distinctive |
| wsuguest40 | 667 aroma is different from 691 and 630 .these two are pungent while 667 is milder | wsuguest56 | i findit too strong than the other two |
| wsuguest5 | the other two were the same and very strong. this smelled similar but not nearly as strong | wsuguest20 | Smells like roasted broccoli |
| wsuguest53 | it smelled like bleach, the others smelled sulphur-ish | wsuguest68 | it smelled like old broccoli, instead of a pool. |
| wsuguest77 | two have garlic aroma to me, while 541 doesnt have | wsuguest13 | much stronger aroma than the other two |
| wsuguest71 | It is very different from the other two samples, it has a bleach-like aroma | wsuguest15 | This one smelled strongly of roasted celery and the other two were very weak. |
| wsuguest11 | There is hardly an aroma with this sample while the others have a strong base smell. | wsuguest25 | The other two samples didn't have such an intense smell. They primarily smelled like salty water. 630 was foul and pungent. |
| wsuguest4 | This sample did not smell anything like the first 2 samples. | wsuguest51 | remarkably strong. i wanted to put 541 which was remarkably faint. (and i suppose 667 was remarkably unremarkable) |
| wsuguest66 | I chose 667 because it didn't have a strong egg smell like the other two samples have. | wsuguest2 | 667 and 541 smell like cleaning products. |
| wsuguest70 | 667 smells less bad, it has a chlorine scent but the other two smell like if an onion was capable of farting. | wsuguest43 | * |
| wsuguest78 | it is milder | wsuguest7 | smelled like gasoline mixed with sulfur while the other two smelled more like chlorine and sulfur |
| wsuguest52 | 691 and 630 smelled like cabbage. 667 smelled like chlorine | wsuguest61 | Very strong, garlic scent |
| wsuguest76 | chlorine smell as opposed to a sulfur smell | wsuguest75 | it had a strong smell of artificial garlic/onion while the others smelled like pool water |
| wsuguest23 | the other two smelled like roasted garlic, this one did not. | wsuguest1 | strength of aroma |
| wsuguest35 | Less of an odor than the other two | wsuguest19 | It stood out and had a very distinct aroma that was different from the first two. |
| wsuguest34 | There was an alcohol-like smell in 667, the other two had a skunk-like pungency to them | wsuguest3 | different smell then other two |
| wsuguest58 | 667 smelled the most like gasoline or fuel. The other two samples 691 and 630 smelled more like brussel sprouts or something more almost flavorful. | wsuguest8 | smelled like rotting broccoli. |
| wsuguest29 | it feels something less intense but not sure | wsuguest74 | it has a strong garlic aroma i comparison to the other two that smell more like chlorine or water. |
| wsuguest10 | The other two samples smelled like a certain hot food while this sample smelled like water | wsuguest57 | more sour |
| wsuguest60 | the aroma of the other two samples are more stimulating and unpleasant. | wsuguest67 | The last one was VERY stinky! |
| wsuguest41 | the smell was milder than the other two | wsuguest9 | this has garlic smell |
| wsuguest42 | it smelled different | wsuguest38 | Other 2 smell of acid |
| wsuguest54 | smelled like propane | wsuguest44 | 667 and 541 kind of smell like natural gas- i have no clue what the other smells like |
| wsuguest28 | It didn't smell as strong. | wsuguest14 | It smelled like gasoline or ethanol, and the other two like brussel sprouts |
| wsuguest64 | the other two were FUNKY but 667 was NOT | wsuguest26 | They all smell really sharply but there's a note of something else in this one. Something really disgustingly bitter, like something rotting in the garbage. Terrible. |
| wsuguest59 | lighter aroma | wsuguest33 | Highest pungency in odor. |
| wsuguest36 | not as strong | wsuguest63 | 630 smelled really bad |
| wsuguest22 | Definitely a lighter, more chlorine, less foul, spoiled milk odor than the other two, which were not good. | wsuguest37 | The sample was much stronger than the other 2 |
| wsuguest65 | The other two smelled like an old onion/savory, while 541 smelled like old pool water. | wsuguest62 | much stronger aroma |
| wsuguest45 | smelled eggy other two smelled like chlorine | wsuguest31 | garlic |
| wsuguest39 | oily gas ! | wsuguest55 | Very strong cooking gas ike smell |

**Supplemental Table S5.** Comments of panelists on why they chose the sample (indole treated and untreated) to be different in the triangle test. These comments are only from those consumers who correctly identified the different sample.

| Panelist # | Comments | Panelist # | Comments |
| --- | --- | --- | --- |
| wsuguest11 | The aroma of this sample has a light scent and seems to smell of an alcohol while the other samples smell of cooking seasonings. | wsuguest21 | all three have odors but 792 appears different |
| wsuguest70 | It smells like chlorine, while the other two smell like raw sewage. | wsuguest79 | It is definitely different to me - smells chlorinated |
| wsuguest23 | the other two smelled like moth balls, this one was slightly onion-y | wsuguest19 | I felt like 499 and maybe 403 smelled a little like bleach, whereas 792 did not. |
| wsuguest40 | because the aroma of 403 is different from 179 and 792 | wsuguest9 | faint garlic smell |
| wsuguest24 | 403 seemed stronger but it was hard to tell. | wsuguest33 | All the three samples had a pungent smell but the sample 792 had a bit less pungent smell. |
| wsuguest4 | this sample did not smell anything like the first 2 samples. | wsuguest13 | It did not have very much aroma (if any), whereas the other two had a simuliar aroma. |
| wsuguest53 | it smelled like bleach. The other two smelled less "clean" | wsuguest45 | smelled like a sharpie marker the others smelled like beef jerky and chlorine |
| wsuguest42 | had a different harsh aroma | wsuguest61 | It is more sour smelling, sharper. |
| wsuguest66 | had a very pungent odor, very chemically. The other two samples were more mellower, and were not as chemically smelling | wsuguest73 | This one smelled like bacterial growth on an agar plate and the other two smelled like bleach |
| wsuguest58 | 403 was an unbearable fuel like smell | wsuguest26 | 403 and 499 had a very strong bleach smell. 179 also smells like bleach but it's muddled with something else, like motor oil. |
| wsuguest52 | 403 is different | wsuguest49 | The sample had more like paint thinner smell, while the other two had different smell. |
| wsuguest29 | I think that has similar aroma but much lesser strength | wsuguest31 | very faint |
| wsuguest77 | there is some petroleum-like smell. | wsuguest37 | Stronger scent than the other two. Also a lot more off putting |
| wsuguest41 | Sample smelled the worst | wsuguest50 | The other 2 smelt like bleach. |
| wsuguest5 | the other two barely had a smell but this one almost smelled like hydrogen peroxide | wsuguest25 | It's aroma was stanky and rancid. It was also the only aroma that like was sharp and left an unpleasant lingering sensation in my nose, mouth, and part of my throat. YUCK |
| wsuguest72 | it smelled like antique shop and the other two smelled like new house | wsuguest51 | Not as chlorine-like. Maybe more earthy. |
| wsuguest34 | 403 had a very sharp unpleasant smell. | wsuguest20 | Smells less intense |
| wsuguest47 | Sample 499 smells like a craft supply store while samples 792 and 179 smell like my friend's house - moth balls and whatever she used to eradicate mice. | wsuguest8 | cause it was the most different |
| wsuguest54 | the other two samples had a distinct pungent smell | wsuguest43 | * |
| wsuguest28 | It smelled stronger than the other two. | wsuguest55 | Totally different smell and less strong |
| wsuguest35 | smelled a bit more ammonia like | wsuguest62 | lack of aroma |
| wsuguest60 | 403 is much stronger and stimulating than the other two. | wsuguest27 | 792 had a very overpowering lilac smell - extremely floral. The last two had a grainy AND floor cleanser smell. What I mean by that is that it smells like when I walk into a working brewery! It's a mix of soaking grain and cleaning/sanitizing solution. I got a *bit* of floral on 403, but the sanitizing solution really overpowered it - maybe because my nose was reeling from 499? 499 smells kind of rubbery too. |
| wsuguest78 | stronger, and particularly unpleasant | wsuguest2 | smells like water. the other 2 smell like bleach |
| wsuguest48 | It smelled like bleach and the other two didn't | wsuguest80 | It smelled more like mothballs than the other two. |
| wsuguest46 | It is the one that smells like straight chlorine/bleach. The other two have something added to it. | wsuguest56 | it is most pungent |
| wsuguest76 | chlorine smell as opposed to a sulfur smell | wsuguest10 | Its has a much stronger smell than the other two. |
| wsuguest6 | This s ample smelled more of ammonia | wsuguest16 | Just seemed a little different. |
| wsuguest30 | smelled wrse than the other 2 | wsuguest69 | mild |
| wsuguest71 | Smells very different, less bad than the other two | wsuguest74 | it smells like nail polish remover while the others smell like water |
| wsuguest12 | This sample had a stronger smell then the other two samples. | wsuguest15 | They all smelled like plastic but the other two smelled also like squeaker toys and 792 was more muted. |
| wsuguest68 | 179 didn't have much of a scent, while the other two smell like chemicals. | wsuguest39 | i feel like i am sitting on the tons of cardboard,reminds me of furniture factory. |
| wsuguest75 | the smell reminded me of a barnyard/wet hay and the other 2 smelled of pool water |  |  |
